# Comprehensive genetic testing improves the clinical diagnosis and medical management of pediatric patients with isolated hearing loss

**DOI:** 10.1186/s12920-022-01293-x

**Published:** 2022-06-27

**Authors:** Jiale Xiang, Yuan Jin, Nana Song, Sen Chen, Jiankun Shen, Wen Xie, Xiangzhong Sun, Zhiyu Peng, Yu Sun

**Affiliations:** 1 College of Life Sciences, University of Chinese Academy of Sciences, Beijing, 100049 China; 2 BGI Genomics, BGI-Shenzhen, Shenzhen, 518083 China; 3 Department of Otorhinolaryngology, Union Hospital of Tongji Medical College, Huazhong University of Science and Technology, Wuhan, 430022 China

**Keywords:** Isolated hearing loss, Genetic testing, Syndromic hearing loss, Nonsyndromic hearing loss mimics

## Abstract

**Purpose:**

Genetic testing is widely used in diagnosing genetic hearing loss in patients. Other than providing genetic etiology, the benefits of genetic testing in pediatric patients with hearing loss are less investigated.

**Methods:**

From 2018–2020, pediatric patients who initially presented isolated hearing loss were enrolled. Comprehensive genetic testing, including *GJB2/SLC26A4* multiplex amplicon sequencing, *STRC/OTOA* copy number variation analysis, and exome sequencing, were hierarchically offered. Clinical follow-up and examinations were performed.

**Results:**

A total of 80 pediatric patients who initially presented isolated hearing loss were considered as nonsyndromic hearing loss and enrolled in this study. The definitive diagnosis yield was 66% (53/80) and the likely diagnosis yield was 8% (6/80) through comprehensive genetic testing. With the aid of genetic testing and further clinical follow-up and examinations, the clinical diagnoses and medical management were altered in eleven patients (19%, 11/59); five were syndromic hearing loss; six were nonsyndromic hearing loss mimics.

**Conclusion:**

Syndromic hearing loss and nonsyndromic hearing loss mimics are common in pediatric patients who initially present with isolated hearing loss. The comprehensive genetic testing provides not only a high diagnostic yield but also valuable information for clinicians to uncover subclinical or pre-symptomatic phenotypes, which allows early diagnosis of SHL, and leads to precise genetic counseling and changes the medical management.

**Supplementary Information:**

The online version contains supplementary material available at 10.1186/s12920-022-01293-x.

## Introduction

Childhood hearing loss affects 1 to 5 per 1,000 newborns [1]. It is acknowledged that the first 36 months after birth represent a critical period in cognitive and linguistic development [1]. At least 60% of childhood bilateral sensorineural hearing loss is due to genetic causes, of which 80% are nonsyndromic with isolated hearing loss, and 20% are syndromic with other abnormalities [2, 3]. To date, over 100 genes are reported to associate with nonsyndromic hearing loss (NSHL), and about 500 genes are associated with syndromic hearing loss (SHL) [4].

Early genetic diagnosis of SHL would significantly reduce other testing and provide opportunities for early intervention [5]. However, it is of great challenged in clinical settings because hearing loss is one of the most heterogeneous conditions. Many outpatients initially present isolated hearing loss and phenotypes other than hearing loss are subtle or late-onset. Moreover, some subtle phenotypes are hard to be identified in pediatric patients by otolaryngologists [6]. The SHL masquerading as NSHL is called NSHL mimics.

The SHL genes can be identified in NSHL patients due to genetic heterogeneity [6]. Of 102 NSHL probands without a causative variant in known NSHL genes, Bademci and co-authors identified four patients having pathogenic variants in SHL genes [7]. However, the characters of enrolled patients in this study were unknown. In pediatric patients with isolated hearing loss, the prevalence of SHL and NSHL mimics should be higher because some phenotypes are late-onset. Recently, two review articles reported that their unpublished data revealed the NSHL mimics comprise up to 25 – 30% of all genetic diagnoses in children with apparent NSHL [5, 8]. However, without clinical follow-up and examinations, it is unclear if these children would develop syndromic hearing loss.

With the advent of next-generation sequencing techniques, a growing number of clinical laboratories implemented genetic testing (hearing loss panel or exome sequencing) to uncover the genetic etiology of hearing loss [9]. Consensus recommendations from the International Pediatric Otolaryngology Group suggested comprehensive genetic testing for children with bilateral NSHL [10].The diagnostic yield of genetic testing for NSHL patients ranged from 40 to 65% among different studies [10], depending on the method used and ethnic background of patients. Other than the genetic etiology, the benefits of genetic testing in pediatric patients are less investigated, which requires clinical follow-up.

In this study, we enrolled 80 pediatric outpatients who initially presented isolated hearing loss and were clinically diagnosed as NSHL. Our comprehensive genetic testing achieved a high definitive diagnostic yield (66%) and likely diagnostic yield (8%). With the aid of genetic testing, following clinical follow-up and examinations, the clinical diagnosis of 11 (19%) molecular diagnosed patients was altered from NSHL to SHL or NSHL mimics. More importantly, they were referred to new specialists and more specific genetic counseling and medical management was conducted.

## Material and methods

This study was approved by the institutional review boards at Tongji Medical College of Huazhong University of Science and Technology.

### Pediatric patient recruitment

Between 2018 and 2020, pediatric patients who met the following criteria were recruited at Tongji Medical College of Huazhong University of Science and Technology: (1) outpatients who initially presented isolated hearing loss at the Department of Ear Nose & Throat; (2) bilateral hearing loss; (3) prelingual onset.

### Genetic testing

Considering the significant contribution of the *GJB2* and *SLC26A4* gene in prelingual hearing loss in the East Asian population [11], the two genes were firstly analyzed via a multiplex PCR amplicon sequencing assay [12].When suspected, a *GJB6* deletion analysis was suggested using low-pass genome sequencing [13]. Because *STRC/OTOA* plays a significant role in moderate hearing loss [14], these patients without a genetic diagnosis from the *GJB2* or *SLC26A4* gene were referred to *STRC/OTOA* analysis (SALSA® MLPA® P461 DIS probe mix kit, MRC-Holland, Amsterdam, the Netherlands). Patients with severe or profound hearing loss or negative for *STRC/OTOA* analysis were referred to exome sequencing. Exome sequencing was completed using KAPA HyperExome Probes (Roche, Pleasanton, CA, USA) accompanied by 100-bp paired-end sequencing on an MGISEQ-2000 platform (BGI-Wuhan, Wuhan, China). CNVs were called from WES data using ExomeDepth software version 1.0.7.18 [15]. The initial BAM files and base quality scores were realigned and recalibrated, respectively. After that, the final BAM files used for CNV prediction computation were generated. The hg19 reference was the used for alignment. The criteria evaluated for determining a CNV using this software comprised at least two consecutive altered exons in a region as a minimum cut-off number and a score higher than 50 for the reliability of an actual result.

Sequence variants were interpreted based on the expert specifications of variant interpretation guidelines for genetic hearing loss [16]. Specifically, a semi-automated variant interpretation platform, VIP-HL was used for variant interpretation [17]. 13 out of 24 ACMG/AMP rules, namely PVS1, PS1, PM1, PM2, PM4, PM5, PP3, BA1, BS1, BS2, BP3, BP4, and BP7, were automated activated based on aggregated information from external databases. While case/segregation (PM3, PS2/PM6, PS4, PP1, PP4, BS4, BP2, and BP5), and functional (BS3 and PS3) criteria were manually curated.

All reported sequence variants were confirmed via Sanger sequencing (single nucleotide variants), qPCR (exon-level copy number variations (CNV)), or low-pass genome sequencing (subchromosomal CNVs). Family segregation analysis was performed.

The positive genotype was defined as follows: (1) patients harboring pathogenic/likely pathogenic variants consistent with the inheritance pattern and segregating with hearing loss was defined as definitive diagnosis; (2) patients with pathogenic/likely pathogenic variants in trans with a variant of uncertain significance in autosomal recessive condition was defined as likely diagnosis.

### Clinical follow-up and diagnosis

When variants in SHL genes were identified in patients with isolated hearing loss, clinical follow-ups were conducted. Patients were recalled for further clinical examinations, which included but were not limited to, audiometry, physical examination, otoscopy, ophthalmoscopy, developmental assessment, and electrocardiogram analysis. The clinical follow-up ended in September 2021.

Finally, SHL was clinically diagnosed when phenotypes other than the hearing loss were uncovered by the date of clinical follow-up. If the genotypes were reported to be intensively associated with syndromic conditions in public literature, which did not present in the pediatric patients by the date of clinical follow-up, we define these patients as NSHL mimics.

## Results

### Cohort characteristics

Between January 2018 and December 2020, a total of 80 patients with isolated, prelingual, bilateral hearing loss were enrolled. Overall, 73% (n = 58), 7% (n = 6), 19% (n = 15), 1% (n = 1) of patients were diagnosed with profound, severe, moderate, and mild sensorineural hearing loss, respectively. Most patients (85%, 68/80) reported no family history of hearing loss. Of 73 patients who received the newborn hearing screening at birth, 88% (64/73) were referred for audiological evaluations, and 12% (9/73) passed the hearing screening program (Additional file 2: Table S1). By the date of follow-up, 83% (66/80) patients received hearing aids or cochlear implants.

### Genetic diagnosis

**Fig. 1:**
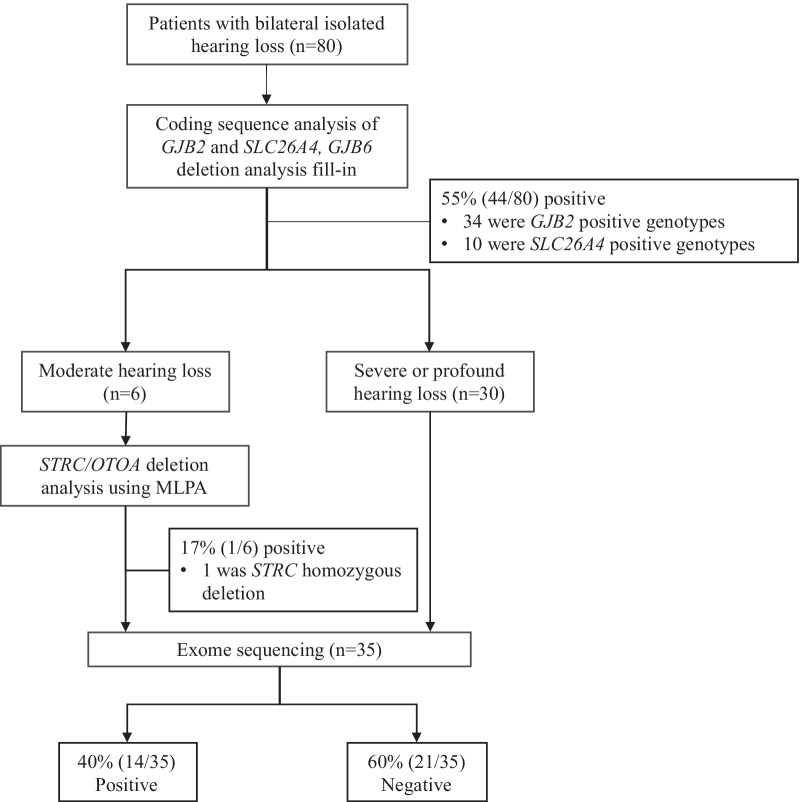
The flow diagram of the comprehensive genetic testing. MLPA, multiplex ligation-dependent probe amplification. Positive, definitive diagnosis and likely diagnosis

With a hierarchical genetic testing strategy, the definitive genetic etiology was confirmed in 53 out of 80 (66%) pediatric patients and likely genetic etiology was confirmed in 6 out of 80 (8%) (Fig. 1 and Additional file 3: Table S2). Specifically, 55% (44/80) patients had positive genotypes in the *GJB2* or *SLC26A4* gene. Of note, one child was diagnosed as a compound heterozygote of NM_004004.6(*GJB2*):c.299_300delAT and del(*GJB6*-D13S1854) (Additional file 1: Figure S1). Six patients with moderate hearing loss were negative for *GJB2* and *SCL26A4* sequencing. They were referred to *STRC/OTOA* analysis. As a result, a homozygous deletion in the *STRC* gene was identified in one (17%, 1/6) child (Additional file 1: Figure S2). The remaining 35 undiagnosed patients were referred to exome sequencing. Exome sequencing revealed the definitive and likely genetic etiology for 40% (14/35) patients. At the end, 26% (21/80) patients remain genetically undiagnosed.

Of 59 genetically definitive and likely diagnosed patients, CNVs were identified in 5 (8%) patients, including one subchromosomal CNV (P10), one *GJB6* deletion (P11), one *STRC* homozygous deletion (P59), two exon-level CNVs (P54 and P62). Five (8%) patients harbored 7 novel variants, which were not reported either in literature or public databases. The autosomal recessive inheritance and autosomal dominant inheritance accounted for 86% (51/59) and 14% (8/59), respectively (Additional file 3: Table S2).

### Genetic testing improves clinical diagnosis and medical management

**Fig. 2:**
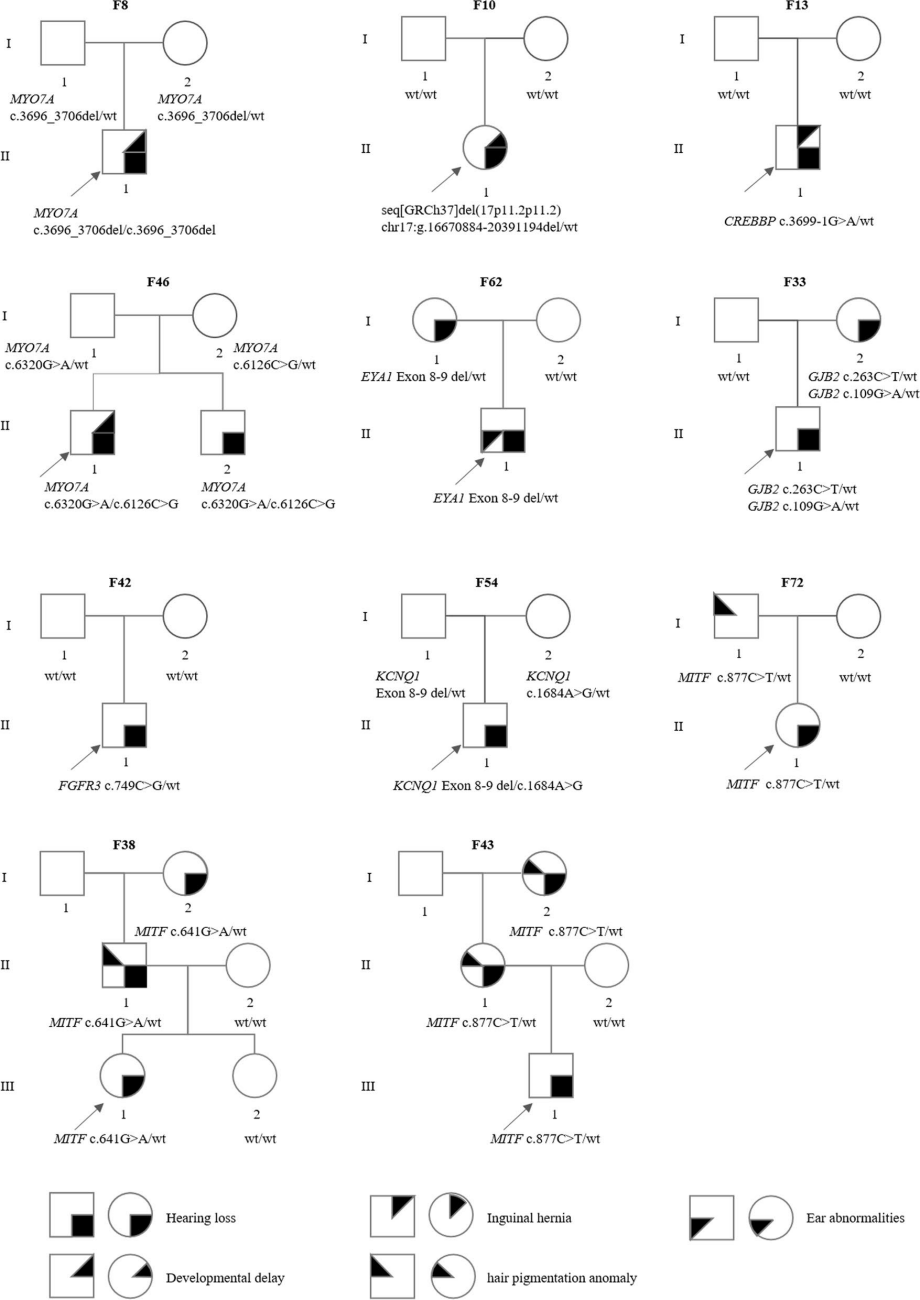
Pedigree diagnosed with syndromic or nonsyndromic hearing loss mimics. The probands are pointed by arrows, squares indicate males, and circles females. Phenotypes are defined as shown below. F, Family, del, deletion, wt, wild type

Prior to genetic testing, the enrolled pediatric probands presented isolated hearing loss, which led to the clinical diagnosis of NSHL. However, genetic testing identified patients harboring variants in genes associated with SHL. These patients were referred to new specialists, and clinical follow-up and examinations were performed. As a result, phenotypes other than hearing loss were identified in five patients (P8, P10, P13, P46, and P62), suggesting a clinical diagnosis of SHL (Fig. 2 and Table 1). By the date of follow-up, six patients (P33, P38, P42, P43, P54, and P72) still presented isolated hearing loss, suggesting NSHL mimics. To sum up, genetic testing altered the clinical diagnosis in 19% (11/59) of genetically diagnosed pediatric patients (Table 2).

**Table 1:** Clinical follow-up and examinations of patients clinically diagnosed with syndromic or nonsyndromic mimics hearing loss

Proband	Clinical diagnosis	Gene	Allele 1	Allele 2	Disease	Severity of HL	Age at latest follow-up (m)	Clinical follow-up and examinations	Management change
P8	SHL	*MYO7A*	c.3696_3706del	c.3696_3706del	Usher syndrome, type 1B	profound	42	Ophthalmoscopy, no abnormality	Annual ophthalmologic evaluation
								Peabody Developmental Motor Scales, mild developmental delay	
P10	SHL	Multiple genes	seq[GRCh37]del(17p11.2 p11.2) chr17:g.16670884-20391194del	-	Smith-Magenis Syndrome	moderate	35	Revised Gesell Developmental Schedules: Developmental delay	Referral to new specialist
P13	SHL	*CREBBP*	c.3699-1G>A	-	Menke-Hennekam syndrome 1	profound	53	Ultrasound inspection, Inguinal hernia	Surgery management
P46	SHL	*MYO7A*	c.6320G>A	c. 6126C>G	Usher syndrome, type 1B	profound	23	Ophthalmoscopy, no abnormality	Annual ophthalmologic evaluation
								Peabody Developmental Motor Scales, mild developmental delay	
P62	SHL	*EYA1*	Exon 8–9 del	-	Branchiootic syndrome	severe	53	Physical examination, preauricular pits, mild cup-shaped ears, small right ear	External ear development monitoring
P33	NSHL mimics	*GJB2*	c.263C>T and c.109G>A	-	Keratitis-ichthyosis-deafness syndrome	profound	25	Physical examination, no abnormality	Referral to new specialist
P38	NSHL mimics	*MITF*	c.641G>A	-	Waardenburg syndrome, type 2A	profound	28	Physical examination, no abnormality	Referral to new specialist
P42	NSHL mimics	*FGFR3*	c.749C>G	-	Muenke syndrome	moderate	26	Physical examination, no abnormality	Referral to new specialist
P43	NSHL mimics	*MITF*	c.877C>T	-	Waardenburg syndrome, type 2A	profound	30	Physical examination, no abnormality	Referral to new specialist
P54	NSHL mimics	*KCNQ1*	c.1684A>G	Exon 8–9 del	Jervell and Lange-Nielsen syndrome	profound	18	Electrocardiography, no abnormality	Paddles for defibrillation during cochlear implantation surgery; cardiac monitoring
P72	NSHL mimics	*MITF*	c.877C>T	-	Waardenburg syndrome, type 2A	profound	15	Physical examination, no abnormality	Referral to new specialist

P, Proband, m, month, HL, hearing loss, SHL, syndromic hearing loss, NSHL mimics, nonsyndromic hearing loss mimics

**Table 2:** Clinical diagnosis of 59 genetically diagnosed patients based on genetic testing results

Disease	OMIM number	Gene	Inheritance	No	Percentage (%)
Nonsyndromic hearing loss				48	81
Deafness, autosomal recessive 1A	220290	*GJB2*	AR	33	56
Deafness, autosomal recessive 4, with enlarged vestibular aqueduct	600791	*SLC26A4*	AR	10	17
Deafness, autosomal recessive 7	600791	*TMC1*	AR	2	3
Deafness, autosomal recessive 3	600316	*MYO15A*	AR	1	2
Deafness, autosomal recessive 16	603720	*STRC*	AR	1	2
Deafness, autosomal recessive 12	601386	*CDH23*	AR	1	2
Syndromic hearing loss				5	8
Smith-Magenis Syndrome	182290	*Multiple genes*	AD	1	2
Menke-Hennekam syndrome	180849	*CREBBP*	AD	1	2
Usher syndrome type 1B	600060	*MYO7A*	AR	2	3
Branchiootic syndrome	602588	*EYA1*	AD	1	2
Nonsyndromic hearing loss mimics				6	10
Waardenburg syndrome, type 2A	193510	*MITF*	AD	3	5
Keratitis-ichthyosis-deafness syndrome	148210	*GJB2*	AD	1	2
Muenke syndrome	602849	*FGFR3*	AD	1	2
Jervell and Lange-Nielsen syndrome	220400	*KCNQ1*	AR	1	2
Total		–		59	100

AD, autosomal dominant, AR, autosomal recessive

### Syndromic hearing loss

In proband 8, a frameshift variant (c.3696_3706del) in *MYO7A* was identified in a homozygous state. In proband 46, two nonsense variants (c.6320G>A and c.6126C>G) in *MYO7A* were identified in compound heterozygous *in trans*. *MYO7A* is associated with Usher syndrome, type 1B (OMIM #276900), an autosomal recessive disorder characterized by retinitis pigmentosa, vestibular dysfunction, and hearing loss [18]. Clinical examinations via ophthalmoscopy revealed no abnormalities of retinitis pigmentosa in both two probands. However, via the Peabody Developmental Motor Scales [19], mild delayed motor development was confirmed at 42 months and 23 months in proband 8 and proband 46, respectively.

In proband 10, a 3.7 Mb heterozygous deletion in the chromosome 17p11.2 region was detected by exome sequencing, which was confirmed by low-pass genome sequencing. The 17p11.2 deletion causes Smith-Magenis syndrome (OMIM# 182290). The clinical features include distinctive craniofacial and skeletal features, global developmental delay, cognitive impairment, mental retardation, as well as hearing loss [20]. Further clinical examinations were counseled. Through the development assessment based on Revised Gesell Developmental Schedules [21], the mild developmental delay was clinically confirmed at the age of 17 months, which was not initially noticed by clinicians and parents.

In proband 13, exome sequencing detected a novel splicing variant in *CREBBP* (c.3699-1G>A). The loss of function variant of *CREBBP* is causative for Rubinstein-Taybi syndrome (OMIM# 180849) or Menke-Hennekam syndrome 1(OMIM# 618332) [22]. Further clinical examinations revealed that the proband lacks the facial and limb dysmorphism associated with Rubinstein-Taybi syndrome. Moreover, ultrasound inspection revealed a condition of inguinal hernia at the age of 4 years, which was consistent with the external genital abnormalities in individuals with Menke-Hennekam syndrome 1. After the counseling and physician assessment, a surgical repair of inguinal hernias was performed.

In proband 62, a novel heterozygous variant (Exon 8–9 del) in *EYA1* was identified. It was inherited from his mother and maternal grandmother. Branchiootic syndrome 1 (OMIM #602588) caused by *EYA1* variants showed a variable spectrum of manifestations, including hearing loss, branchial fistulae, preauricular pits, and renal abnormalities [23]. Although the parents declared no family history of other phenotypes except for hearing loss, a thorough clinical evaluation was conducted according to the genetic testing results. At the age of 4 years, a manifestation of slight preauricular pits, milder cup-shaped ears, and a small right ear was uncovered in the proband, consistent with the phenotype of Branchiootic syndrome 1. No further treatment or surgery was taken after the counseling because the phenotype is subtle.

### Nonsyndromic hearing loss mimics

Variants related to SHL genes were identified in six probands (P33, P38, P42, P43, P54 and P72), whereas clinical phenotypes other than hearing loss were not observed, indicating a clinical diagnosis of NSHL mimics (Fig. 2 and Additional file 2: Table S1).

More specifically, two linked heterozygous variants (c.109G>A and c.263C>T) in *GJB2*, a de novo heterozygous variant in *FGFR3* (c.749C>G), a heterozygous variant in *MITF* (c.877C>T) were identified in proband 33, 42, and 72 respectively. The reported variants caused Keratitis-ichthyosis-deafness (KID) syndrome (OMIM #148210), Muenke syndrome (OMIM #602849), and Waardenburg syndrome, type 2A (OMIM #193510), respectively. However, the three probands did not have additional clinical phenotypes after clinical examinations by the time of the latest follow-up.

Additionally, two variants in compound heterozygote in *KCNQ1* (c.1684A>G and Exon 8–9 del) were identified by exome sequencing in proband 54. *KCNQ1* is associated with Jervell and Lange-Nielsen syndrome (JLNS, OMIM# 220400), which is characterized by profound congenital hearing loss and a prolonged QTc interval in the electrocardiography waveforms [6]. Electrocardiography detected a normal QTc interval in proband 54, suggesting NSHL mimics. Concerning life-threatening cardiac arrhythmias in JLNS patients [24], paddles for defibrillation were prepared in the process of cochlear implantation surgery at the age of 1 year. Regular cardiac monitoring was counseled for the prevention of sudden cardiac death.

In proband 38 and 43, a missense variant (c.641G>A) and a nonsense variant (c.877C>T) and in *MITF* were identified by exome sequencing, respectively, which led to the genetic diagnosis of Waardenburg syndrome, type 2A (OMIM# 193510). The missense variant in proband 38 was inherited from the hearing-impaired father and paternal grandmother. However, only the father demonstrated premature graying of hair during clinical follow-ups. The c.877C>T was identified in P43. Clinical follow-up revealed that the mother and maternal grandmother had a slight white forelock. The family members did not consider the abnormal pigmentation as a severe phenotype, thus did not report to clinicians in the recruitment.

## Discussion

Childhood hearing loss is one of the most heterogeneous conditions. Early identification of hearing loss and understanding its etiology can assist with the prognosis and counseling of families [25]. In this study, we developed a comprehensive genetic testing approach, which identified the definitive genetic etiology for 66% (53/80) of pediatric patients, and the likely genetic etiology for 8% (6/80) of pediatric patients. More importantly, we uncovered that at least 19% of genetically diagnosed patients who were initially presenting isolated hearing loss were SHL or NSHL mimics. Benefit from the genetic testing and counseling, these patients were referred to new specialists for further assessment and regular monitoring, which ended the diagnostic odyssey.

Our tiered-based genetic testing strategy has a definitive and likely diagnostic yield of 74% in pediatric patients with prelingual hearing loss. It is higher than the previously reported rate ranging from 40 – 65% [10]. This might be attributable to high proportion of severe and profound patients (80%) in the study cohort. Another explanation is the existence of hotspot variants of the *GJB2* and *SLC26A4* gene in the East Asian population [11] and the exon-level and subchromosomal CNV analysis in our analyzing pipeline. In this study, 5/59 (8%) of genetically diagnosed patients were contributable to CNVs, including one subchromosomal CNV, one *GJB6* deletion compound with a *GJB2* variant, one *STRC* homozygous deletion, two exon-level CNVs. Nevertheless, the genetic spectrum reinforces the genetic heterogeneity of hearing loss, and a comprehensive genetic testing approach including exome sequencing, MLPA, low-pass genome sequencing is warranted.

SHL gene are commonly identified in children presenting with isolated hearing loss. Two review articles reported their unpublished data [5, 8]. Specifically, Shearer et al. reported that NSHL mimics comprise up to 25% of all genetic diagnoses in children [5]. Vona et al. reported that up to 30% of *GJB2*-mutation-negative children who are clinically identified as NSHL harbor pathogenic variants in genes associated with SHL [8]. However, without clinical follow-up and examinations, it is unknown if these patients would have subclinical or pre-symptomatic phenotypes. Herein, we reported that 19% of genetically diagnosed pediatric patients were clinically diagnosed SHL or NSHL mimics after genetic testing and clinical follow-up and examinations. Nevertheless, these data imply that a considerable portion of pediatric patients who initially present with NSHL would gradually develop SHL with age. More importantly, with the aid of genetic testing, early diagnosis prior to the presence of clinical phenotypes is feasible. Genetic testing provides valuable information for counseling and medical management and would significantly reduce other testing and provide opportunities for early intervention [5].

We wish to stress that the clinical diagnosis of a specific condition is of great challenge, particularly in a gene associated with NSHL and SHL. To not exaggerate the proportion of SHL and NSHL mimics, we only counted patients whose genotype is strongly associated with syndromic phenotypes in public literature. Following this rule, patients with pathogenic variants in *CDH23* and *SLC26A4* did not be counted in SHL, and they were conservatively categorized in NSHL. *CDH23* is associated with Usher syndrome type ID (USH1D, OMIM #601067) and nonsyndromic autosomal recessive deafness 12 (OMIM #601386). Patients with a truncated mutation (nonsense, frameshift, or splice-site variation) are mostly associated with USH1D, whereas those with missense mutations usually appear to be nonsyndromic [26, 27]. However, USH1D caused by missense mutations was also reported [28]. Therefore, proband 61 harboring two missense variants (c.719C>T and c.7198C>G) in *CDH23* were considered as NSHL in our study.

*SLC26A4* is another good example to demonstrate the complexity. Mutations of *SLC26A4* cause Pendred syndrome (OMIM #274600) and nonsyndromic autosomal recessive deafness 4 (OMIM #600791). Pendred syndrome is distinguished by the presence of thyroid abnormalities which are incompletely penetrant, adolescence onset, and partially influenced by nutritional iodine intake [29, 30]. To date, it is unclear why the same genotype would develop different phenotypes in different patients. Given all the ten children with positive genotypes in *SLC26A4* had normal thyroid by the date of follow-up, we conservatively considered them as NSHL in our study. However, regular monitoring of the thyroid function was still suggested, which may be abnormal during adolescence [31]. Taken together, these results demonstrated the complexity of apparent isolated hearing loss, and longitudinal genotype–phenotype association studies are urgently needed.

Another important issue is to understand the underlying mechanisms of NSHL mimics. One possible explanation is that clinical phenotypes of a given syndrome are not yet present or not easily evaluated by otolaryngologists at the time when NSHL was diagnosed. For example, children diagnosed with Usher syndrome may manifest retinitis pigmentosa many years after the onset of hearing impairment [8]. Two children (P8 and P46) in our cohort were genetically diagnosed with Usher syndrome. By the latest follow-up, ophthalmoscopy revealed no abnormalities of retinitis pigmentosa in these patients. Regular ophthalmologic examinations to monitor the progression of the condition were counseled to the children’s guardians. Another example was the identification of JLNS caused by *KCNQ1* in P54. Individuals with JLNS can have significant cardiac events in early childhood [6]. JLNS is thought to be one of the causes of sudden infant death syndrome. Although the measurement of electrocardiography was normal in P54, avoiding physical or emotional exertion was suggested. Additionally, subtle manifestations such as mild developmental abnormality confirmed in P8, P10 and P46, are prone to be ignored by otolaryngologists.

Variable penetrance might be another explanation for NSHL mimics. The same pathogenic variant associated with SHL may show incomplete penetrance and present differently between different people. Three patients (P38, P43 and P72) were genetically diagnosed with Waardenburg syndrome without additional clinical manifestations. However, the relatives harboring the same pathogenic variant displayed mild abnormal pigmentation of the hair. These variable manifestations within the same families suggest a variable penetrance of these variants and the phenotypic heterogeneity [32].

Another case of the effect of variable penetrance is the diagnosis with P33. The proband had a heterozygous c.263C>T (p.Ala88Val), in cis with an AR pathogenic variant (p.Val37Ile), in *GJB2*, which inherited from his nonsyndromic hearing-impaired mother. Further exome sequencing eliminated other possible causes of nonsyndromic dominant deafness. Previous studies have demonstrated c.263C>T is associated with Keratitis-ichthyosis-deafness (KID) syndrome, and may cause infant early lethality [33]. In our study, the proband and his mother only showed isolated hearing loss. This case represents a significant departure from what is known about KID syndrome. A similar situation has been reported with another lethal KID syndrome mutation in *GJB2*, p.Gly45Glu. The pathogenic effect of p.Gly45Glu can be confined by the presence of another heterozygous nonsense mutation p.Tyr136Ter, leading to healthy phenotype [34]. Likewise, the pathogenic effect of p.Ala88Val was possibly confined by the AR pathogenic variant or other cis-regulatory variation [35], and showed an incomplete penetrance. In vivo expression analysis and In vitro function validations of the mutant allele are warranted.

In conclusion, syndromic hearing loss and nonsyndromic hearing loss mimics are common in pediatric patients who initially present with isolated hearing loss. The comprehensive genetic testing provides not only a high diagnostic yield but also valuable information for clinicians to uncover subclinical or pre-symptomatic phenotypes, which allows early diagnosis of SHL, and leads to precise genetic counseling and changes the medical management.

## Supplementary Information

**Additional file 1: Figure S1.** Pedigree with GJB6 deletion. **Figure S2.** Pedigree with STRC homozygous deletion.

**Additional file 2: Table S1.** Phenotypic and genotypic characteristics of patients evaluated in this study.

**Additional file 3: Table S2.** The genetic diagnosis results of 80 patients.

## Data Availability

The data used and/or analyzed during the current study are available from the corresponding author on reasonable request. The data are not publicly available due to privacy or ethical restrictions.
