# Supplementary material for: Comprehensive genetic testing improves the clinical diagnosis and medical management of pediatric patients with isolated hearing loss
Source: BMC Med Genomics. 2022 Jun 27;15:142. doi: 10.1186/s12920-022-01293-x (PMC9235092; doi:10.1186/s12920-022-01293-x)
Supplement: Supplementary file 1 — Additional file 1: Figure S1. Pedigree with GJB6 deletion. Figure S2. Pedigree with STRC homozygous deletion. [file 12920_2022_1293_MOESM1_ESM.docx]

**Supplemental**


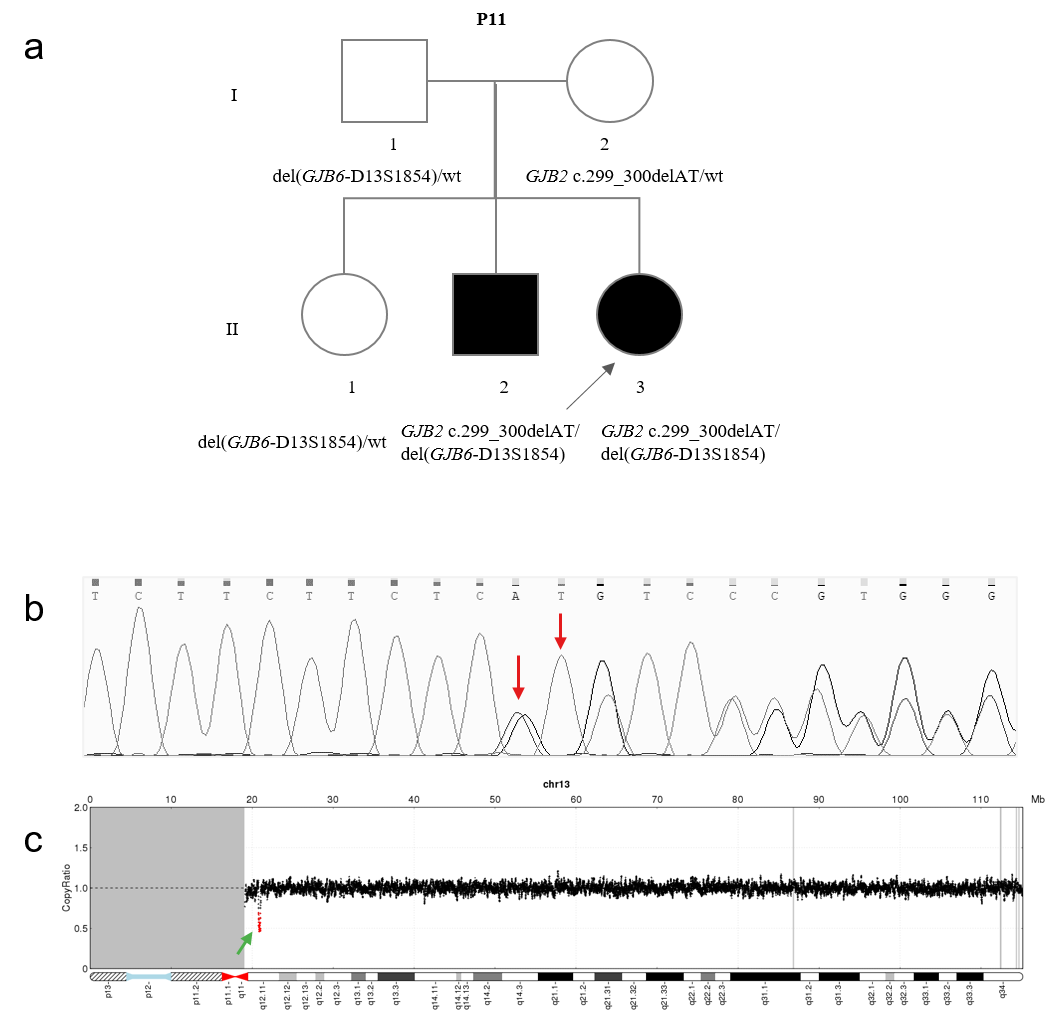


**Figure S1 Pedigree with *GJB6* deletion**

(a) Pedigree information. Black and white symbols each represent affected and normal individuals. The proband had one hearing loss brother and a normal hearing sister, we tested all individuals and confirmed the co-segregation of the family, the affected brother carried compound heterozygous variants, the unaffected sister carried heterozygote variant (b) Sanger confirmation testing result of c.299_300delAT of *GJB2*, the red arrow indicates this variant. (c) The del(GJB6-D13S1854) (seq[GRCh37] del(13q12.11q12.11) chr13:g.20797693-21034825) is represented by a green arrow identified by low-pass genome sequencing. del, deletion, wt, wild type.


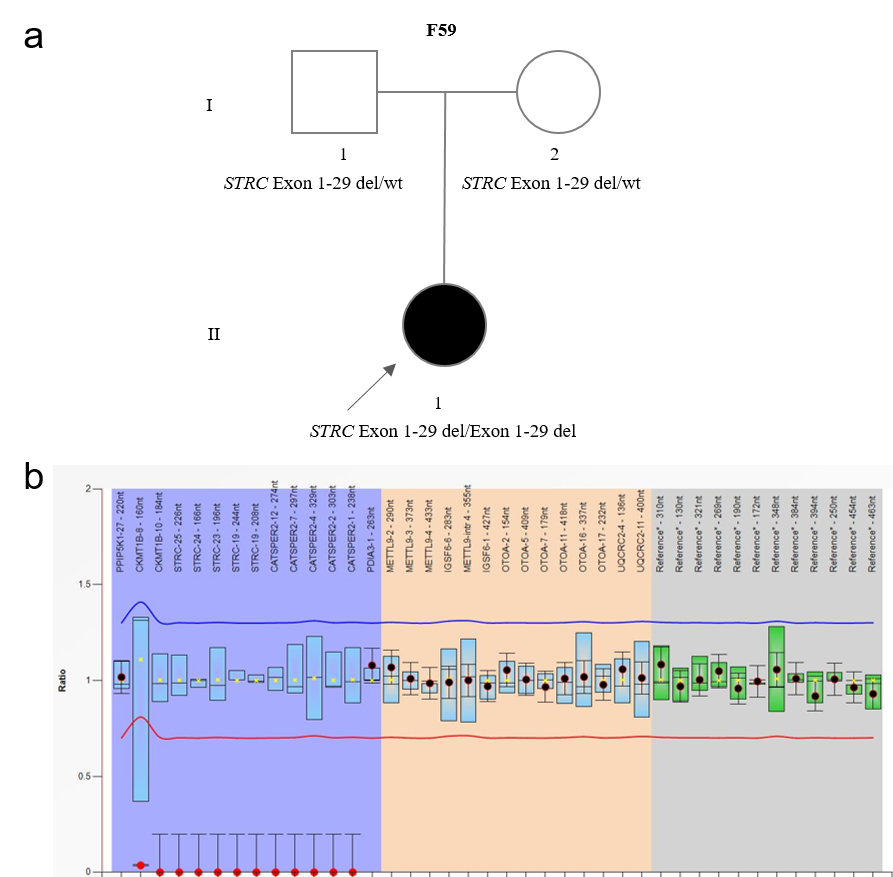


**Figure S2 Pedigree with *STRC* homozygous deletion**

(a) Pedigree information. Black and white symbols each represent affected and normal individuals. (b) MLPA result of *STRC*. The red dot with 0 copy ratio indicated homozygous deletion of *STRC*. del, deletion, wt, wild type
