# Supplementary material for: Comprehensive genetic testing improves the clinical diagnosis and medical management of pediatric patients with isolated hearing loss
Source: BMC Med Genomics. 2022 Jun 27;15:142. doi: 10.1186/s12920-022-01293-x (PMC9235092; doi:10.1186/s12920-022-01293-x)
Supplement: Supplementary file 2 — Additional file 2: Table S1. Phenotypic and genotypic characteristics of patients evaluated in this study. [file 12920_2022_1293_MOESM2_ESM.docx]

**Table S1.** **Phenotypic and genotypic characteristics of patients evaluated in this study.**

| **Characteristic** | **No.** | **Percentage** |
| --- | --- | --- |
| **All** | 80 | 100% |
| **Sex** |  |  |
| Male | 47 | 59% |
| Female | 33 | 41% |
| **Family history** |  |  |
| Yes | 12 | 15% |
| No | 68 | 85% |
| **Newborn hearing screening** | |  |
| Pass | 9 | 11% |
| referral | 64 | 80% |
| Not tested | 7 | 9% |
| **Age of audiology evaluation** | |  |
| ≤3 months | 16 | 20% |
| 3-30 months | 64 | 80% |
| **Severity** |  |  |
| Mild | 1 | 1% |
| Moderate | 15 | 19% |
| Severe | 6 | 7% |
| Profound | 58 | 73% |
| **CT or MRI** |  |  |
| Abnormal | 9 | 11% |
| Normal | 21 | 26% |
| Not tested | 50 | 63% |
| Hearing aids or cochlear implants |  |  |
| Yes | 66 | 83% |
| No | 14 | 18% |

Severity: Mild 26–40 dB, Moderate 41–60 dB, Severe 61–80 dB, Profound >80 dB, CT computed tomography, MRI magnetic resonance imaging.
